# Assessment of the Body Composition and Parameters of the Cardiovascular Risk in Juvenile Idiopathic Arthritis

**DOI:** 10.1155/2015/619023

**Published:** 2015-03-09

**Authors:** Ewa Jednacz, Lidia Rutkowska-Sak

**Affiliations:** Paediatric Clinic of Rheumatology, Institute of Rheumatology, Spartanska 1, 02-637 Warsaw, Poland

## Abstract

The study was aimed to evaluate cardiovascular risk parameters, body mass index (BMI) centiles for sex and age, and body fat percentage using the electric bioimpedance method in children with juvenile idiopathic arthritis (JIA). 30 children with JIA participated in the study. A control group included 20 children. Patients were well matched for the age and sex. The body mass and body fat percentage were determined using the segmental body composition analyser; the BMI centiles were determined. All patients had the following parameters determined: lipid profile, hsCRP, homocysteine, and IL-6. The intima media thickness (IMT) was measured. Patients with JIA had significantly lower body weight, BMI, and the BMI centile compared to the control group. The IL-6 levels were significantly higher in patients with JIA compared to the control group. There were no differences between two groups with regard to the lipid profile, % content of the fat tissue, homocysteine levels, hsCRP, and IMT. Further studies are necessary to search for reasons for lower BMI and BMI centile in children with JIA and to attempt to answer the question of whether lower BMI increases the cardiovascular risk in these patients, similarly as in patients with rheumatoid arthritis (RA).

## 1. Introduction

Atherosclerosis is a disease known from many years [1, 2]. There were many theories regarding the aetiology of atherosclerosis, but groundbreaking was the theory of Russel Ross, who announced that atherosclerosis is an inflammatory disease [3]. Currently, apart from commonly known risk factors predisposing to atherosclerosis development more attention has been paid to new risk factors such as acute phase proteins like CRP, proinflammatory cytokines, homocysteine, and noninvasive methods to assess the intima media thickness (IMT), the values of which correlate with the advancement of atherosclerosis.

Clinical consequences of an atherosclerotic process are present in the adult population; nonetheless, atherosclerotic lesions start to form in early childhood, even in the foetal life [4, 5]. In 2011, the American Academy of Pediatrics published extensive guidelines on lowering the cardiovascular risk in children and adolescents [6]. High and medium cardiovascular risk groups were distinguished. The medium risk group includes children with chronic inflammatory diseases such as systemic lupus erythematosus and juvenile idiopathic arthritis (JIA). JIA is the most common chronic arthropathy of the developmental age that develops with arthritis, extra-articular lesions, and systemic complications. Diagnostic criteria include a disease onset prior to the age of 16 years, duration of symptoms for at least 6 weeks, and exclusion of other causes of arthritis based on the so-called exclusion list [7]. In contrary to the population of adults with rheumatoid arthritis (RA), there is little data indicating an increased cardiovascular risk in children with JIA. Patients with RA have a shorter life expectancy compared to the general population. Cardiovascular diseases are the main causes of death in this population [8–10]. In 2009, The European League against Rheumatism (EULAR) presented its recommendations regarding screening tests for circulatory diseases in patients with RA and other forms of arthritis and according to them, RA is a disease associated with a higher risk of cardiovascular disease development [11]. Studies of recent years have provided more and more evidence that there is a correlation between inflammatory processes in the course of RA and development of atherosclerotic lesions. The synovial membrane in RA and atherosclerotic plaques have pathological similarities. Similar mechanisms in the synovial membrane in RA and in the development of atherosclerotic plaques include T and B cells, macrophages, adhesive molecules, proinflammatory cytokines: tumor necrosis factor (TNF) alpha, interleukin 1 (IL-1), interleukin 6 (IL-6), and chemokines [12].

The study was aimed to evaluate cardiovascular risk parameters, body mass index (BMI) centiles for sex and age, and body fat percentage using the electric bioimpedance method in children with JIA.

## 2. Materials and Methods

### 2.1. Study Population

30 children with JIA who were patients of the Paediatric Clinic of Rheumatology, Institute of Rheumatology in Warsaw, participated in the study between September 2012 and April 2013. Inclusion criteria were as follows: the age between 11 and 17 years and the diagnosis of JIA based on the criteria of the International League of Associations for Rheumatology [13]. 16 children suffered from oligoarticular and 14 from polyarticular JIA. Children took only medicines associated with JIA; they did not suffer from any other medical conditions. 22 children received methotrexate (in monotherapy or in combination treatment), 6 children sulfasalazine (in monotherapy or in combination treatment), 5 children chloroquine (in monotherapy or in combination treatment), 2 children cyclosporine A (in combination treatment), 1 child azathioprine (in combination treatment), 5 children TNF inhibitors (in combination treatment), and 8 children glucocorticosteroids (in combination treatment). Children were divided into groups depending on disease duration, below one year, 6 children (20%), and above one year, 24 children (80%), and depending on the disease activity. According to the criteria by Ringold and Wallace, an inactive disease phase was indicated by the following criteria: no joints with active arthritis, lack of fever, rash, serositis, splenomegaly, generalized lymphadenopathy associated with JIA, no signs of active uveitis; normal erythrocyte sedimentation rate (ESR) or C-reactive protein (CRP); and no signs of active disease based on the physician global assessment of disease activity [14]. The criteria of an inactive disease phase were met by 11 children (37%) and those of an active disease by 19 children (67%). A child was diagnosed as overweight when the body mass index (BMI) indicated the value equal to or above the 85th centile and below the 95th centile for the sex and age, whereas obesity was diagnosed when the BMI was equal to or above the 95th centile [15–17]. There were no overweight or obese children in the study group.

A control group included 20 children at the age between 10 and 16 years who were patients at the Paediatric Clinic of Rheumatology and in whom rheumatologic diseases were excluded with appropriate paediatric tests. These children did not take any medicines, were not supervised by any other specialists, and were not overweight or obese.

All patients had a physical examination performed, blood samples were drawn early in the morning after overnight fasting, and anthropometric measurements and the IMT were assessed at the Paediatric Clinic of Rheumatology.

The study was approved by the Bioethics Committee at the Institute of Rheumatology in Warsaw. All parents and children provided their consent for study participation.

### 2.2. Anthropometric Measurements

The body mass and body fat percentage were determined using the segmental body composition analyser, TANITA BC-418 MA, using bioelectric impedance technology, according to the manufacturer's instructions. This model has 4 additional electrodes in hand grips apart from a standard platform with 4 electrodes, which provides a system of 8 electrodes. As a result, it is possible to perform a detailed assessment of separate body segments.

The BMI was determined based on the body mass and height measurements using the following formula: the body mass in kilograms divided by the height in square meters. The BMI centiles were determined using a calculator prepared based on the OLAF project (calculator's author: Anna Manerowska, Warsaw University of Technology). An additional aim of the OLAF project included preparation of standards in a form of centile charts presenting the body mass index with relation to the sex and age of children and adolescents aged 7–18 years, representative for the Polish population [18], whereas the main aim included preparation of arterial pressure standards in a form of centile charts.

### 2.3. Laboratory Measurements

Lipid profiles were determined using dry chemistry methods with the Vitros S 350 analysers by Ortho Clinical Diagnostics (Ortho Clinical Diagnostics Vitros S 350 Chemistry Analyzer), according to the manufacturer's instructions.

High-sensitivity C-reactive protein (hs-CRP) was determined using latex-enhanced immunoturbidimetry with the Cobas C 501 analyser, according to the manufacturer's instructions. Human CRP agglutinates with latex particles that are coated with monoclonal antibodies to human CRP. A precipitate formed is measured turbidimetrically at the wave length of 552 nm.

Homocysteine was measured immunochemically with microparticles and a chemiluminescence marker used for quantitative determination of L-homocysteine in human serum samples using the ARCHITECT i System, according to the manufacturer's instructions. IL-6 was determined using the double-bond Sandwich ELISA method that belongs to immunoenzymatic methods, using a Diagnostics Pasteur device.

### 2.4. Carotid Ultrasonography

The IMT was measured with a semiautomatic method using a linear transducer of the Vivid S5 device by GE Medical Systems. The intima-medial thickness of the common carotid artery 1-2 cm proximally to the bifurcation at the segment of 1 cm [19] was calculated based on automatic detection of outlines of the intima-media contours on the posterior vascular wall.

The following parameters were calculated: mean IMT, maximum IMT, minimum IMT, standard deviation of IMT measurements, and number of successful IMT measurements. Finally, the mean IMT on the right and left was assessed. The test was performed in a supine position, with an abducted head, slightly tilted oppositely to the examined side, and preceded by a 10-minute rest.

### 2.5. Statistical Analysis

Continuous variables were assessed whether they were normally distributed using the Kolmogorov-Smirnov test and presented as means and standard deviations (SD) or medians and interquartile ranges (IQR: 25th and 785th), as appropriate. Comparisons between groups were made using the independent *t*-test, one-way ANOVA (normal distribution), Mann-Whitney *U* test, or Kruskal-Wallis *H*-tests (irregular or skewed distribution). Significance levels were adjusted for multiple comparisons using the Tukey post hoc method. A correlation between continuous variables was performed using Pearson's or Spearman's correlation coefficient (*r*), as appropriate. Categorical variables were expressed as frequencies and percentages, and the Fisher's exact or *χ* ^2^ tests were used for comparison.

A two-tailed *P* value of <0.05 was considered statistically significant. Statistical analyses were performed with SAS 9.2 (Cary, NC, USA).

## 3. Results

Comparison between children with JIA and the control group and the demographic, clinical, laboratory, and ultrasonographic data of patients with JIA and healthy controls are shown in Table 1. Patients were well matched for the age and sex. Patients with JIA had significantly lower body weight (*P* = 0.0066), BMI (*P* = 0.0302), and the BMI centile (*P* = 0.0308) compared to the control group. The IL-6 levels were significantly higher in patients with JIA compared to the control group (*P* = 0.0137). There were no differences between two groups with regard to the lipid profile, % content of the fat tissue, homocysteine levels, hsCRP, and IMT.

The analysis in patients with active and inactive JIA and the control group (Table 2) presents a comparison between patients with active and inactive JIA and the control group.

Patients with active JIA had higher hsCRP levels compared to patients with inactive disease (*P* = 0.0154, inactive versus active JIA: 0.0109). There were no differences between patients with active and inactive disease with regard to lipid parameters, BMI centile, % of the fat tissue, homocysteine levels, and IMT assessment. The analysis in oligoarticular and polyarticular JIA and the control group (Table 3) presents a comparison of patients with oligoarticular and polyarticular JIA and the control group. There were no significant differences between patients with oligoarticular and polyarticular JIA with regard to lipid parameters, BMI centile, % of the fat tissue, hsCRP levels, IL-6 levels, homocysteine levels, and IMT assessment.

### 3.1. Correlations

Table 4 presents correlations between hsCRP, IL-6, homocysteine levels, IMT and lipid parameters, BMI, and BMI centile in all studied patients with JIA. A positive correlation between the patient age and homocysteine levels was observed (*r* = 0.35; *P* = 0.06), but it was not statistically significant. A positive correlation between the homocysteine levels and LDL cholesterol levels was observed (*r* = 0.22; *P* = 0.25) and between the homocysteine levels and IL-6 levels (*r* = 0.35; *P* = 0.14), but they were not statistically significant. There were no correlations between other analysed parameters.

## 4. Discussion

Atherosclerosis is an inflammatory disease. Obesity is one of commonly known cardiovascular risk factors. American studies demonstrated obesity in 18% of patients with JIA [20]. In German studies 15% of children were overweight and 7% were obese [21]. In patients with RA special attention is paid to the fact that low BMI is associated with cardiovascular risk [22]. In RA patients rheumatoid cachexia is observed. It is characterised by the loss of body mass, but fat mass tends to be maintained or increased [23]. Less is known about assessing of the body composition in patients with JIA. In a longitudinal study using whole body dual X-ray absorptiometry scans the percentage of total body fat was greater in patients with JIA and total lean body mass was less in patients with JIA compared to healthy children [24, 25]. But in a study with adult patients with JIA, who were in remission or with active disease, patients with JIA had significantly less body fat than healthy controls [26]. Some researchers draw attention to the fact that JIA patients exhibit impaired nutrition and lower BMI values compared to healthy children [27, 28]. Factors associated with this fact are not clearly determined, and they may be associated with an inflammatory process, secreted cytokines, absorption disturbances, effects of medicines [29], inadequate dietary intake, and physical activity limitation [30]. Patients with systemic JIA have increased expenditure of energy [31]. In our studies patients with JIA exhibited lower BMI centiles for sex and age compared to the control group, and this difference was statistically significant, but there was no significant difference between the fat tissue percentages. We used the bioelectric impedance method as it is noninvasive, simple, and quick to perform, even though dual-energy X-ray absorptiometry (DEXA) is more accurate to assess fat mass and lean body mass. Further studies are necessary to determine whether a low BMI centile in children with JIA is associated with a cardiovascular risk, which has been demonstrated for patients with RA and low BMI.

Patients with RA have dyslipidemia defined as higher levels of total cholesterol and/or triglycerides and/or lower HDL levels. Dyslipidemia depends on the disease activity, and the higher the disease activity score (DAS) is, the lower total cholesterol levels are; however, the HDL fraction levels are reduced to a larger extent; therefore the atherogenicity index is increased [32]. Studies in children with JIA demonstrate varied results [33–35]. Some of them indicate lack of significant differences in a lipid profile compared to healthy children, and it was also observed in our study.

Endothelial dysfunction is observed at an early stage of atherosclerosis. Proinflammatory cytokines, such as IL-6, are involved in this process and they increase the ICAM-1 expression and CRP synthesis [36]. The administration of IL-6 to mice susceptible to atherosclerosis and to mice resistant to atherosclerosis receiving a cholesterol-rich diet resulted in atherosclerotic lesions developing only in mice susceptible to atherosclerosis [37]. The increased levels of proinflammatory cytokines are observed in RA [38], similarly in children with JIA [39]. In studies on patients with RA, IL-6 played an especially vital role in the vascular endothelial activation [40]. Our study demonstrated the increased IL-6 levels in children with JIA compared to the control group, and this difference was statistically significant.

As it is possible to assay the CRP levels with a high-sensitivity method, it became possible to precisely determine low CRP levels that indicate mild inflammation, assumed to contribute to atherosclerosis pathogenesis. The increased CRP levels are a risk factor for cardiovascular incidents [41]. Our study did not demonstrate differences in the hsCRP levels between children with JIA and the control group; however, there was a statistically significant difference in the hsCRP levels between patients with active and inactive disease.

The elevated homocysteine levels are an independent risk factor for brain stroke and ischaemic heart disease, and when these levels are reduced the risk becomes lower [42]. In 2004, the guidelines were published that concluded that the reference homocysteine levels should be determined for separate populations depending on many factors, such as the age, and also supplementation with folic acid and B group vitamins [43]. The studies by Gonçalves et al. did not demonstrate significantly elevated homocysteine levels compared to healthy children despite treatment with methotrexate, which was probably a result of appropriate supplementation with folic acid [35]. We obtained similar results in our study, and it might stem from the fact that the majority of patients received methotrexate, and all patients receiving methotrexate took folic acid supplementation. A positive correlation between the age and the homocysteine levels (*r* = 0.35; *P* = 0.06) was observed, but it did not reach statistical significance probably due to a low number of patients.

Moreover, positive correlations between the homocysteine levels and LDL cholesterol levels and between the homocysteine levels and IL-6 levels were observed, but they were not statistically significant probably due to a low number of patients.

In the search for noninvasive methods to assess the risk of atherosclerosis, ultrasonography is of interest as it allows determining the intima media thickness. The IMT is a factor predicting the stage of atherosclerosis and it may help assess the cardiovascular risk in asymptomatic patients with a moderate cardiovascular risk [44]. In the studies by Vlahos in children with JIA increased IMT was observed only in children with a systemic disease, and this difference was not observed for oligoarticular or polyarticular form [45]. The studies by Breda demonstrated increased IMT in children with JIA compared to healthy children [46]. Our study did not demonstrate differences in IMT between healthy children and children with JIA. It might have been caused by the fact that the study group did not include children with a systemic disease where the intensity of inflammatory lesions is especially high. Our study has some limitations. It is a cross-sectional study with a relatively low number of patients. The nutritional status assessment performed in order to compare children with JIA with the control group did not consider a diet of children, and everyday diet was not reported, but it may have a significant effect on the nutritional status. We did not consider the functional impairment status and physical activity levels in patients with JIA. Both may influence the nutritional status of the patient. When assessing patients with JIA only oligoarticular and polyarticular disease were considered, and the systemic disease was not taken into account due to a very low number of patients with this form of disease at the time of study enrollment. It is possible that differences in the IMT evaluation or in other cardiovascular risk factors might have been visible in such patients. Additionally, the results of the study were not analysed with regard to the treatment applied, and the treatment itself affects the cardiovascular risk [47].

## 5. Conclusions

As it was demonstrated by our study, the impaired nutritional status may be present in children with JIA. Despite a lower BMI centile the fat tissue percentage was not significantly lower in these children. Further studies are necessary to search for reasons for lower BMI centiles in children with JIA and to attempt to answer the question of whether lower BMI increases the cardiovascular risk in these patients, similarly as in patients with RA.

Further studies with large groups of patients with JIA are required in order to assess the cardiovascular risk in this population.

## Figures and Tables

**Table 1:** Characteristics of patients with JIA and control group.

	JIA group, *n* = 30	Control group, *n* = 20	*P*
Age, years	14,0 ± 1,8	14,4 ± 1,8	0.4301
Female	23 (76,7%)	15 (75,0%)	1.0000
Weight, kg	48,4 ± 8,6	55,5 ± 9,0	0.0066^*^
Height, cm	160,7 ± 8,8	166,0 ± 10,3	0.0592
BMI	18,6 ± 2,2	20,1 ± 2,3	0.0302^*^
BMI percentile	37,2 ± 23,6	52,9 ± 25,6	0.0308^*^
FAT %	19,9 ± 5,8	23,0 ± 5,5	0.0603
Total cholesterol, mg/dL	151,4 ± 25,1	153,4 ± 26,8	0.7928
Triglycerides, mg/dL	89,4 ± 38,8	92,1 ± 37,3	0.8113
LDL cholesterol, mg/dL	77,6 ± 23,6	77,6 ± 23,9	0.9996
HDL cholesterol, mg/dL	58,5 ± 13,7	57,4 ± 14,9	0.7866
hsCRP, mg/L	0,40 [0,30–1,60]	0,45 [0,30–0,70]	0.7342
IL-6, pg/mL	56,0 [0,0–272,2]	0,0 [0,0-0,0]	0.0137^*^
Homocysteine, *µ*mol/L	8,9 ± 2,9	9,2 ± 2,5	0.7327
IMT, mm	0,47 ± 0,04	0,47 ± 0,04	0.9665

BMI: body mass index, % FAT: % body fat, LDL: low density lipoprotein, HDL: high density lipoprotein, hsCRP: high senstivity C-reactive protein, IL-6: interleukin 6, IMT: intima media thickness, and ^*^statistically significant.

**Table 2:** Characteristics of patients with JIA according to disease activity.

	JIA inactive, *n* = 11	JIA active, *n* = 19	Control group, *n* = 20	*P*

Age, years	13,7 ± 1,8	14,2 ± 1,8	14,4 ± 1,8	0.5762
Female	10 (90,9%)	13 (68,4%)	15 (75%)	0.4066
Weight, kg	47,7 ± 10,2	48,7 ± 7,8	55,5 ± 9,0	0.0248 CG versus I: 0.0582 CG versus A: 0.0516
Height, cm	159,3 ± 10,3	161,5 ± 8,1	166,0 ± 10,3	0.1428
BMI	18,6 ± 2,6	18,6 ± 2,0	20,1 ± 2,3	0.0979
BMI percentile	39,6 ± 22,7	35,8 ± 24,6	52,9 ± 25,6	0.0917
FAT %	21,0 ± 5,4	19,3 ± 6,0	23,0 ± 5,5	0.1293
Total cholesterol, mg/dL	156,6 ± 27,8	148,3 ± 23,6	153,4 ± 26,8	0.6770
Triglycerides, mg/dL	92,3 ± 42,6	87,7 ± 37,5	92,1 ± 37,3	0.9266
LDL cholesterol, mg/dL	74,8 ± 21,8	79,2 ± 25,1	77,6 ± 23,9	0.8909
HDL cholesterol, mg/dL	63,4 ± 13,5	55,6 ± 13,4	57,4 ± 14,9	0.3435
hsCRP, mg/L	0,30 [0,20–0,50]	0,70 [0,40–3,90]	0,45 [0,30–0,70]	0.0154^*^ I versus A: 0.0109
IL-6, pg/mL	0,0 [0,0–369,6]	168,4 [0,0–255,9]	0,0 [0,0-0,0]	0.0384^*^ CG versus A: 0.0109
Homocysteine, *µ*mol/L	8,6 ± 2,6	9,1 ± 3,0	9,2 ± 2,5	0.8191
IMT, mm	0,49 ± 0,05	0,46 ± 0,03	0,47 ± 0,04	0.1796

BMI: body mass index, FAT %: % body fat, LDL: low density lipoprotein, HDL: high density lipoprotein, hsCRP: high sensitivity lipoprotein, IL-6: interleukin 6, IMT: intima media thickness, ^*^statistically significant, CG: control group, I: inactive, and A: active.

**Table 3:** Characteristic of patients with JIA according to type of JIA and control group.

	Polyarticular, *n* = 14	Oligoarticular, *n* = 16	Control group, *n* = 20	*P*
Age, years	14,9 ± 1,6	13,3 ± 1,8	14,4 ± 1,8	0.0441
Female	10 (71,4%)	13 (81,2%)	15 (75,0%)	0.8427
Weight, kg	50,6 ± 7,1	46,4 ± 9,5	55,5 ± 9,0	0.0109^*^ P versus O: 0.0083
Height, cm	163,2 ± 5,5	158,4 ± 10,7	166,0 ± 10,3	0.0665
BMI	19,0 ± 2,3	18,3 ± 2,1	20,1 ± 2,3	0.0699, P versus O: 0.0606
BMI percentile	36,5 ± 27,0	37,9 ± 21,0	52,9 ± 25,6	0.0985
FAT %	19,9 ± 6,3	19,9 ± 5,5	23,0 ± 5,5	0.1745
Total cholesterol, mg/dL	145,6 ± 26,2	156,4 ± 23,7	153,4 ± 26,8	0.5022
Triglycerides, mg/dL	78,4 ± 30,0	99,0 ± 43,8	92,1 ± 37,3	0.3293
LDL cholesterol, mg/dL	77,1 ± 25,9	77,9 ± 22,4	77,6 ± 23,9	0.9961
HDL cholesterol, mg/dL	58,2 ± 14,1	58,7 ± 13,9	57,4 ± 14,9	0.9605
hsCRP, mg/L	0,70 [0,40–3,90]	0,40 [0,25–0,55]	0,45 [0,30–0,70]	0.0962
IL-6, pg/mL	150,5 [0,0–239,6]	0,0 [0–369,6]	0 [0,0-0,0]	0.0327 CG versus P: 0.0067
Homocysteine, *µ*mol/L	8,6 ± 2,5	9,2 ± 3,2	9,2 ± 2,5	0.8209
IMT, mm	0,46 ± 0,03	0,48 ± 0,05	0,47 ± 0,04	0.3418

BMI: body mass index, % FAT: % body fat, LDL: low density lipoprotein, HDL: high density lipoprotein, hsCRP: high sensitivity lipoprotein, IL-6: interleukin 6, IMT: intima media thickness, ^*^statistically significant, P: polyarticular, and O: oligoarticular.

**Table 4:** Correlations [Pearson/Spearman correlation coeficient and *P* value: *r*(*P*)] between hsCRP, IL-6, homocysteine, IMT, and cardiovascular risk factors in children with JIA.

JIA all patients, *n* = 30				
	hsCRP	IL-6	Homocysteine	IMT
Age	*r* = 0.29	*r* = 0.21	*r* = 0.35	*r* = −0.06
	*P* = 0.13	*P* = 0.39	*P* = 0.06	*P* = 0.75

BMI	*r* = 0.30	*r* = −0.09	*r* = 0.25	*r* = 0.05
	*P* = 0.10	*P* = 0.71	*P* = 0.19	*P* = 0.79

BMI percentile	*r* = 0.25	*r* = −0.11	*r* = 0.08	*r* = 0.05
	*P* = 0.18	*P* = 0.66	*P* = 0.69	*P* = 0.79

Total cholesterol, mg/dL	*r* = 0.24	*r* = −0.05	*r* = 0.28	*r* = 0.04
	*P* = 0.20	*P* = 0.85	*P* = 0.14	*P* = 0.82

LDL cholesterol, mg/dL	*r* = 0.31	*r* = −0.15	*r* = 0.22	*r* = 0.09
	*P* = 0.10	*P* = 0.53	*P* = 0.25	*P* = 0.64

HDL cholesterol, mg/dL	*r* = −0.11	*r* = 0.19	*r* = −0.06	*r* = 0.07
	*P* = 0.54	*P* = 0.44	*P* = 0.77	*P* = 0.72

hsCRP, mg/L	*r* = 1.00	*r* = −0.07	*r* = 0.32	*r* = −0.10
		*P* = 0.77	*P* = 0.09	*P* = 0.61

IL-6, pg/mL	*r* = −0.07	*r* = 1.00	*r* = 0.35	*r* = −0.27
	*P* = 0.77		*P* = 0.14	*P* = 0.26

Homocysteine, *µ*mol/L	*r* = 0.32	*r* = 0.35	*r* = 1.00	*r* = −0.21
	*P* = 0.09	*P* = 0.14		*P* = 0.26

JIA: juvenile idiopathic arthritis, BMI: body mass index, LDL: low density lipoprotein, HDL: high density lipoprotein, hsCRP: high sensitivity lipoprotein, IL-6: interleukin 6, and IMT: intima media thickness.
